# Furosine, a Maillard Reaction Product, Triggers Necroptosis in Hepatocytes by Regulating the RIPK1/RIPK3/MLKL Pathway

**DOI:** 10.3390/ijms20102388

**Published:** 2019-05-14

**Authors:** Huiying Li, Yizhen Wang, Huaigu Yang, Yangdong Zhang, Lei Xing, Jiaqi Wang, Nan Zheng

**Affiliations:** 1 State Key Laboratory of Animal Nutrition, Institute of Animal Science, Chinese Academy of Agricultural Sciences, Beijing 100193, China; thufit2012@126.com (H.L.); m13613613572_2@163.com (Y.W.); yanghgxms@163.com (H.Y.); zhangyangdong@caas.cn (Y.Z.); lei_xing163@163.com (L.X.); wang-jia-qi@263.net (J.W.); 2 Key Laboratory of Quality & Safety Control for Milk and Dairy Products of Ministry of Agriculture and Rural Affairs, Institute of Animal Science, Chinese Academy of Agricultural Sciences, Beijing 100193, China; 3 Laboratory of Quality and Safety Risk Assessment for Dairy Products of Ministry of Agriculture and Rural Affairs, Institute of Animal Sciences, Chinese Academy of Agricultural Sciences, Beijing 100193, China

**Keywords:** furosine, liver damage, hepatocytes, LPC (18:0), PLA2-3, necroptosis

## Abstract

As one of the typical Maillard reaction products, furosine has been widely reported in a variety of heat-processed food. Though furosine was shown to be toxic on organs, its toxicity mechanism is still unclear. The present study aimed to investigate the toxicity mechanism of furosine in liver tissue. An intragastric gavage mice model (42-day administration, 0.1/0.25/0.5 g/kg of furosine per day) and a mice primary hepatocyte model were employed to investigate the toxicity mechanism of furosine on mice liver tissue. A metabonomics analysis of mice liver, serum, and red blood cells (RBC) was performed. The special metabolic mediator of furosine, lysophosphatidylcholine 18:0 (LPC (18:0)) was identified. Then, the effect of the upstream gene phospholipase A2 gamma (*PLA2-3*) on LPC (18:0), as well as the effect of furosine (100 mg/L) on the receptor-interacting serine/threonine-protein kinase (RIPK)1/RIPK3/mixed lineage kinase domain-like protein (MLKL) pathway and inflammatory factors, was determined in liver tissue and primary hepatocytes. PLA2-3 was found to regulate the level of LPC (18:0) and activate the expression of RIPK1, RIPK3, P-MLKL, and of the inflammatory factors including tumor necrosis factor α (TNF-α) and interleukin (IL-1β), both in liver tissue and in primary hepatocytes. Upon treatment with furosine, the upstream sensor PLA2-3 activated the RIPK1/RIPK3/MLKL necroptosis pathway and caused inflammation by regulating the expression of LPC (18:0), which further caused liver damage.

## 1. Introduction

The Maillard reaction is one of the most important reactions resulting from the heating process. The chemicals generated from amino acids and sugars in the Maillard reaction are named Maillard reaction products (MRPs). As one of the typical MRPs [1], furosine (C_12_H_18_N_2_O_4_, 254.28 g/mol) was identified as ε-*N*-(2-furoylmethyl)-l-lysine in 1968 [2,3]; it is broadly found in a variety of foods including dairy products, cereals, honey, bakery products, etc., and its content is directly related to the degree of heating treatment and to the storage time [4]. Furosine was proved to be one of the most stable derivatives of the Amadori compounds and is also well known as a reliable marker and indicator of the nutritional value of heat-treated food, which always reflects the degree of protein loss and freshness [5,6].

Although many articles have been published about the quantitative detection of furosine especially in milk, the following three questions about furosine are still unclear: First, is furosine toxic? Second, if it is, what organ or tissue is most affected by furosine? Third, what is the toxicity mechanism of furosine? Currently, there are very few available data evaluating the overall toxicity and metabolites of furosine, though it is urgent to assess furosine’s safety and control its production in food. In our recent studies, the overall toxicity of furosine was evaluated. We found that furosine inhibits the viability of HepG2 and Hek293 cells in vitro and the increase in animal body weight and causes toxic effects on mice liver and kidney [7,8]. However, the toxicological mechanism and specific metabolites of furosine in living organisms remain unknown.

In this study, we attempted to investigate the toxicity mechanism of furosine in the liver utilizing mice and primary hepatocytes. A metabonomics analysis was performed to identify the specific metabolic mediator of furosine in mice liver, then the role of upstream genes in regulating this metabolite and the necroptosis RIPK1/RIPK3/MLKL pathway was validated. This study is the first to shed light on the toxicity mechanism of furosine in the liver.

## 2. Results

### 2.1. Furosine Increases the Expressios of Liver Biochemical Indicators

To evaluate furosine’s effect on liver function, mice serum was collected, and six indicators were detected by Elisa kits. The results showed that 42-day consecutive administration of furosine caused changes in the expression of ALT (alanine transaminase), AST (aspartate transaminase), ALP (alkaline phosphatase), γ-GT (γ-glutamyl transferase), TBil (total bilirubin), and LDH (lactate dehydrogenase), which mainly reflect the detoxification capability and the damage level of the liver. The levels of ALT and AST, considered as markers of liver injury [9,10], were sharply increased in furosine-treated groups in a dosage-dependent manner. ALP was reported to be a sensitive index for the clinical diagnosis of liver diseases including obstructive jaundice, cholangitic hepatitis, and liver cancer, in which the excess amounts of ALP, overexpressed by liver cells, could not be evacuated regularly in the biliary tract and entered the blood [11,12]. γ-GT is mainly expressed in bile capillaries and hepatocellular microsomes and reflects the degree of extension of liver lesions and of liver parenchyma injury (chronic hepatitis and hepatocirrhosis). A slight upregulation of γ-GT in the serum is always related to hepatitis and pancreatitis, while a severe increase of γ-GT reflects hepatocirrhosis, liver cancer, and bile ducts cancer [13,14,15]. TBil is utilized in diagnosing hepatic diseases and obstruction of the biliary tract, and its upregulation is tightly related to inflammation, necrosis, and toxicosis of the liver tissue [16,17]. LDH is present in nearly all organs and tissues, especially heart, kidney, and liver, and changes of its expression level tend to reflect the susceptibility to hepatopathy [18,19].

**Figure 1:**
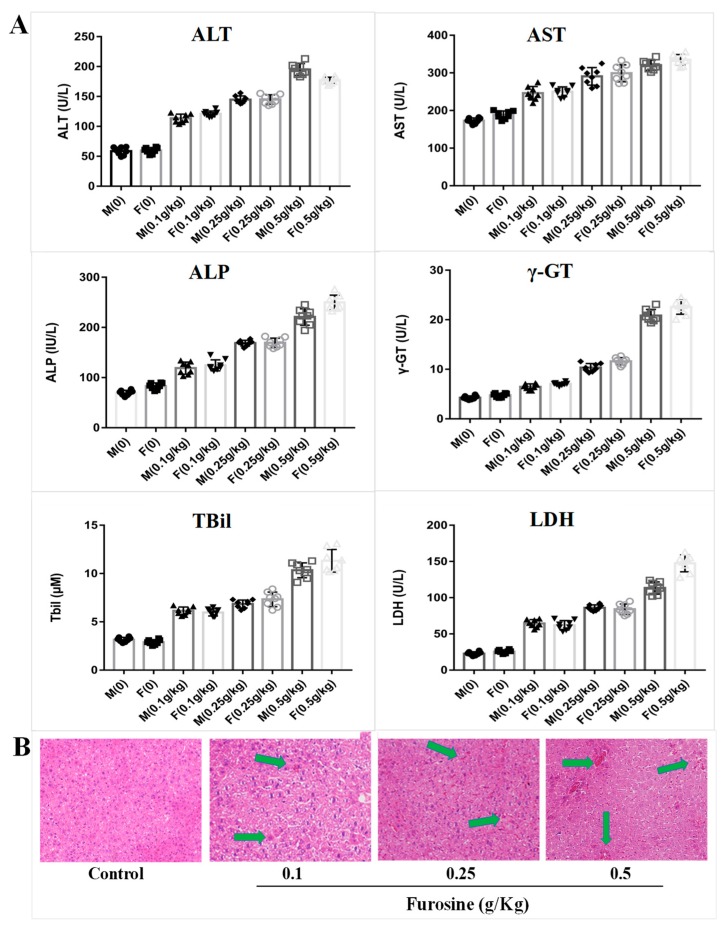
The toxic effect of furosine on mice liver. (**A**) Detection of the biochemical indicators, including alanine transaminase (ALT), aspartate transaminase (AST), alkaline phosphatase (ALP), γ-glutamyl transferase (γ-GT), total bilirubin (TBil), and lactate dehydrogenase (LDH) in mice serum. M: male mouse; F: female mouse. All data are represented as mean ± SD, *n* = 8. (**B**) Liver tissue pathological detection by hematoxylin and eosin (HE) staining. All images were captured under 200× magnification. The green arrows stand for the pathological damages in liver tissue, including cytomorphosis, hemorrhages, etc.

Compared with the control levels, the expression of ALT, AST, ALP, γ-GT, TBil, and LDH in the serum of mice treated with furosine increased at different degrees, and the differences measured were all statistically significant (Figure 1A, *p* < 0.05). These results suggested that long-term administration of furosine caused liver damage and might be related to chronic inflammation, hepatocytes necrosis, and liver toxicosis.

### 2.2. Furosine Exerts and Adverse Effect on the Liver

To evaluate furosine effect on liver morphology, the liver pathological state was analyzed by a histopathological test. The results showed that a 42-day consecutive administration of furosine caused obvious pathological changes in the liver tissue. Compared with the control group, there was a slight edema in the liver tissue of the group treated with 0.1 g/kg furosin, whereas the liver tissue of the mice treated with 0.5 g/kg furosine showed obvious edema and cytomorphosis in some areas, and, occasionally, severe inflammatory cell infiltration and hemorrhages (Figure 1B). The degree of liver injury seemed to be tightly related to the dosage of furosine, indicating a dosage-dependent mechanism of action. The results from the pathological analysis suggested that long-term administration of furosine caused inflammation and pathological changes in liver tissue, which could further lead to hepatocytes toxicosis and liver damage. These results are in accordance with the results of the expression of the biochemical indicators and further confirmed that the liver was the main target organ of furosine, suggesting that several specific metabolites of furosine might be transferred to, produced, or accumulated in the liver.

### 2.3. Transcriptomics Analysis of Furosine Metabolites in Liver Tissue, Serum, and Red Blood Cells

To investigate the toxic mechanism of furosine in the liver, a metabonomics analysis of liver tissue, serum, and red blood cells (RBC) from mice treated with furosine was completed. The results showed that the metabolites detected in mice liver in the furosine treatment groups were obviously different from those found in the control group. By comparing the levels of metabolites between the control group and the three furosine treatment groups, 47 metabolites (withvariable importance in projection (VIP) value ≥ 1) were identified, whose expression levels are reported in the Supplementary data (Supplementary Figure S1).

In the same conditions, metabolites clustering in mice serum and RBC in the furosine treatment groups were different from those detected in the control group. In total, 66 and 12 metabolite candidates were selected from mice serum and RBC, respectively, whose relative expression levels are shown in the Supplementary data (Supplementary Figures S2 and S3).

### 2.4. Screening of Specific Metabolites in Liver Tissue, Serum, and RBC

**Figure 2:**
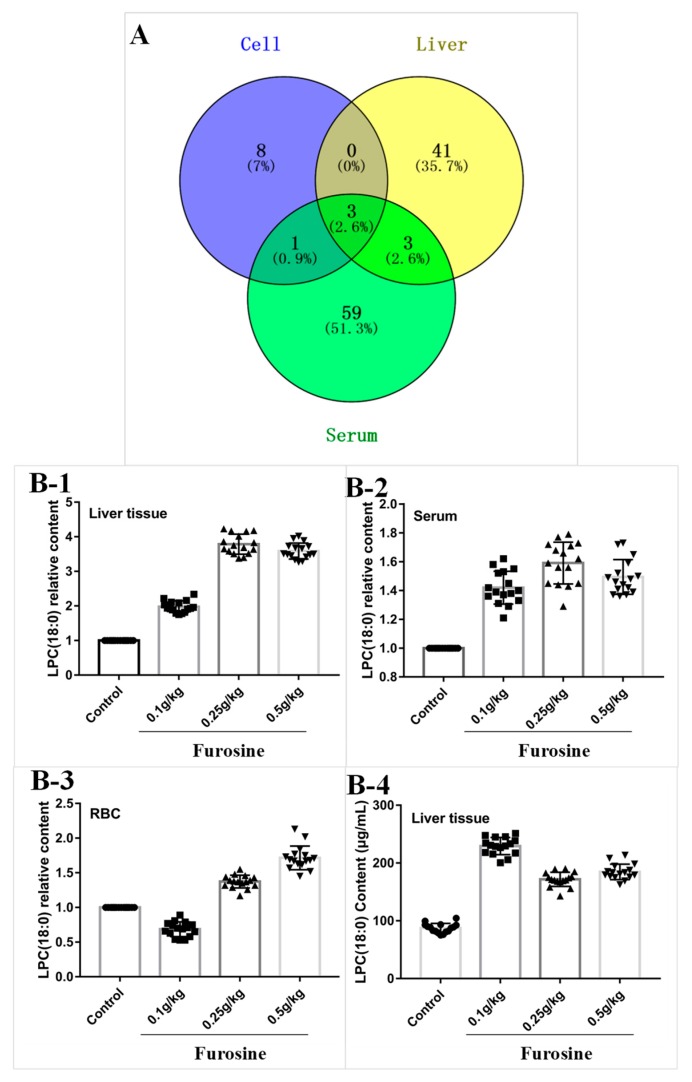
Analysis of different furosine metabolites and detection of the metabolite lysophosphatidylcholine (LPC) (18:0). (**A**) VENN plot. The blue dot stands for metabolites in the red blood cell (RBC) sample, the yellow dot stands for metabolites in liver tissue, the green dot stands for metabolites in serum. The critical metabolites were identified in the overlapping areas. (**B**) LPC (18:0) detection in liver tissue, serum, and RBC. (**B-1**) Relative quantification of LPC (18:0) in liver tissue. (**B-2**) Relative quantification of LPC (18:0) in serum. (**B-3**) Relative quantification of LPC (18:0) in RBC. (**B-4**) Absolute quantification of LPC (18:0) in liver tissue. All the data are represented as mean ± SD, *n* = 16.

By combining the data derived from the analysis of metabolites in the different samples, three common metabolites were identified in a VENN diagram (Supplementary metabolites data), i.e., lysophosphatidylcholine (LPC) (18:0), lysophosphatidylethanolamine (LPE) (18:0), and lysophosphatidylcholine (LPC) (16:0), which showed significant upregulation in the liver, serum, and red blood cells compared with the control (Figure 2A). Interestingly, the three chemicals have a similar structure, especially LPC (18:0) and LPC (16:0), suggesting that the lysophosphatidylcholine family might be tightly related to the toxic effects of furosine. According to accurate quantitative results (Figure 2B), we found that LPC (18:0) increased significantly in the furosine-treated groups compared with the control groups, especially in the liver, validating that LPC (18:0) was the toxic metabolite derived from furosine causing liver injury.

### 2.5. Furosine Enhanced the Expression of PLA2-3, RIPK-1, RIPK-3, P-MLKL, TNF-α, and IL-1β in Liver Tissue and Primary Hepatocytes

In order to detect the specificity of our metabonomics results and clarify the molecular mechanism of the toxicity of furosine, the expression of phospholipase A2 gamma (PLA2-3), related to the synthesis of LPC (18:0), of the necroptosis-related proteins receptor-interacting serine/threonine-protein kinase (RIPK-1, RIPK-3), and mixed lineage kinase domain-like protein (MLKL), and of the inflammatory factors TNF-α and IL-1β was analyzed.

**Figure 3:**
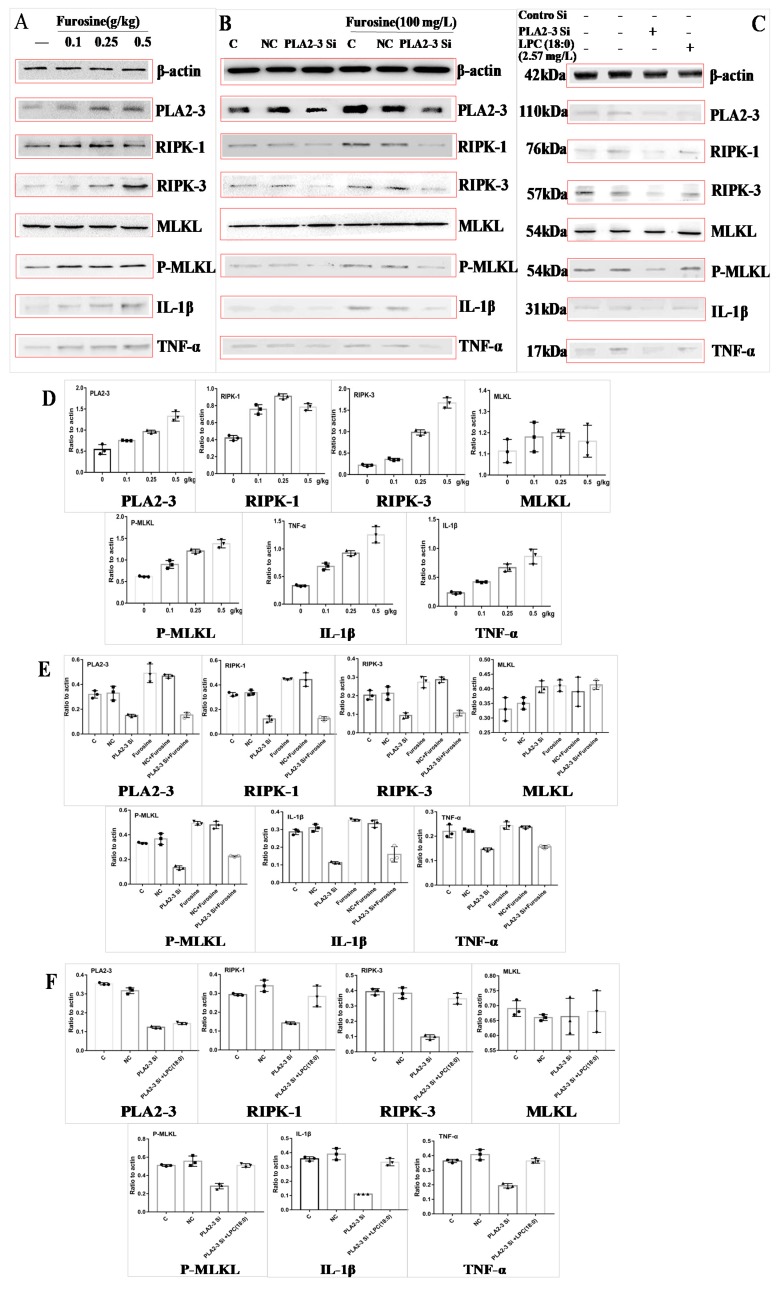
Expression of phospholipase A2 gamma (PLA2-3), receptor-interacting serine/threonine-protein kinase (RIPK-1, RIPK-3), mixed lineage kinase domain-like protein (MLKL), phospho (P)-MLKL, IL-1β, and TNF-α at the protein level (western blotting) and related quantitative data analysis (expression ratio with respect to the actin level). (**A**) Expression of these proteins affected by furosine in liver tissue. (**B**) Expression of these proteins affected by PLA2-3 siRNA and furosine in primary hepatocytes. (**C**) Expression of these proteins affected by PLA2-3 siRNA and LPC (18:0) in primary hepatocytes. (**D**) Densitometric quantitation after normalization relative to β-actin (%) in (**A**). (**E**) Densitometric quantitation after normalization relative to β-actin (%) in (**B**). (**F**) Densitometric quantitation after normalization relative to β-actin (%) in (**C**). Data are expressed as mean ± SD, *n* = 3.

We found that the protein expression levels of PLA2-3, RIPK-1, RIPK-3, P-MLKL, TNF-α, and IL-1β in the liver tissue of mice treated with furosine increased (Figure 3A,D) in a dose-dependent manner, suggesting that furosine could upregulate the expression of PLA2-3 and activate the necroptosis pathway.

By determining cell viability with the CCK-8 kit, the proper dosage (100 mg/L, associated with about 90% survival of primary hepatocytes) of furosine was determined for further tests (data not shown). Upon treating primary hepatocytes with PLA2-3 siRNA, the protein levels of PLA2-3, RIPK-1, RIPK-3, P-MLKL, TNF-α, and IL-1β increased compared with control cells; after the addition of furosine, the levels of these factors did not change compared with those of the PLA2-3 siRNA group (Figure 3B,E), indicating that PLA2-3 could induce hepatocytes necroptosis and inflammation.

LPC (18:0) was added to PLA2-3 siRNA-treated cells to evaluate its role in regulating downstream factors. We found that LPC (18:0) upregulated the protein expression of RIPK-1, RIPK-3, P-MLKL, TNF-α, and IL-1β (Figure 3C,F) compared with the PLA2-3 siRNA group, without affecting the level of PLA2-3 that remained much lower than the control level, thus confirming that PLA2-3 was the upstream regulator of LPC (18:0).

We also found that the mRNA levels of these factors, PLA2-3, RIPK-1, RIPK-3, TNF-α, and IL-1β, was upregulated compared with the control levels (*p* < 0.05) (Supplementary Figure S4).

## 3. Discussion

Furosine is largely present in heat-treated food and is a common early-stage indicator and stable derivative of the Maillard reaction [20]; however, very few data analyzing the toxicity mechanism of furosine in animals are available. Because of the lack of experimental data about the genotoxicity of furosine, the tolerable daily intake (TDI) and the maximum furosine levels acceptable in foods have not been established [21]. The elucidation of furosine effects on health, in relation not only to chronic diseases but also to its toxic targets, could prompt researchers working on optimizing food production to minimize the formation of furosine and obtain safe food products. This work is an initial step in the toxicological assessment of furosine and identified a novel furosine critical metabolite and related pathway.

Biochemical indicators detection always reflects the condition of organ functions. The expression levels of ALT, AST, ALP, γ-GT, TBil, and LDH in mice serum proved that furosine caused toxic effects on the liver. The upregulation of these indicators in our research was consistent with Li’s results, which showed that a 12-week oral administration of pure carboxymethyl lysine (CML, a furosine analogue) at 60 mg/kg resulted in hepatic toxicity [22]. These results in combination with those of HE staining further verified that furosine accumulated and was metabolized in liver tissue, inducing cytomorphosis and blood vessel rupture and evoking inflammation and tissue edema. Therefore, we chose liver as the specific target organ to carry out further research.

Metabonomics analysis is an effective analytical method for the quantitative and qualitative detection of small-molecular-weight metabolites. The overall changes in the levels of endogenous small-molecule metabolites in cells or organisms can be analyzed by metabonomics. Compared with proteomics and genomics, metabonomics analyzes the physiological and pathological changes in an organism or cells by a more comprehensive method [23,24,25,26]. In the present study, the metabolism of liver tissue, serum, and RBC was analyzed. The results suggested that the metabolic state of liver tissue, serum, and RBC of mice was significantly different in furosine-treated group compared with the control group. In particular, some endogenous small-molecule metabolites, i.e., LPC (18:0), LPE (18:0), and LPC (16:0) were identified. In addition, by further accurate quantification of the three metabolites in the samples, LPC (18:0) was found to be the critical metabolite downstream of furosine, suggesting that the main toxicity of furosine on the liver might be associated with it. LPCs is a lipid mediator with pro-inflammatory and pro-atherogenic activities, which is released from apoptotic cells or produced under inflammatory or ischemic conditions as a result of enhanced PLA2 activity [27,28]. LPC family members, especially LPC (18:0), were proved to be a critical factor underlying cardiovascular diseases [29], pneumonia [30], Alzheimer’s disease [31], hypobaric hypoxia [32], hepatitis B [33], and several pathological conditions that are associated with elevated LPC levels in the circulation [34,35,36]. LPCs have a broad spectrum of pro-inflammatory activities, including promotion of cell growth [37], migration [38,39], secretion of chemokines and cytokines [40], generation of reactive oxygen species [41], and upregulation of adhesion molecules, such as ICAM-1, VCAM-1, and selectins [42].

PLA2 family, a growing protein family that comprises cytosolic, secreted, and membrane-associated enzymes [43], includes at least three cytosolic PLA2 (cPLA2) isozymes, nine low-molecular-weight secretory PLA2 (sPLA2) isozymes, several splicing variants of Ca^2+^-independent PLA2 (iPLA2), etc. [44,45,46,47,48,49,50,51,52,53]. cPLA2 γ (PLA2-3) was identified as an ortholog of cPLA2 α and is a key enzyme in eicosanoid production. PLA2-3 was reported to be located in the endoplasmic reticulum (ER) and mitochondria and to have lysophospholipase activity beside PLA2 activity. The synthesis of LPC (18:0) is also regulated by PLA2-3 to some degree, and down-regulation of LPC by inhibiting PLA2-3 might be helpful to alleviate or even cure several diseases related to LPC [54,55,56]. On the basis of the above studies and q-PCR detection, we found the level of PLA2-3 increased significantly in furosine-treated liver tissue compared with the control group, which confirmed that activation of PLA2-3 increased the expression of LPC (18:0) in mice.

A pair of kinases, RIPK-1 and RIPK3, as well as the RIPK3 substrate MLKL, cause a form of programmed necrotic cell death in mammals, termed necroptosis. Necroptosis is caused by the TNF family of cytokines [57,58] in response to the activation of TNF receptor family members; RIPK1 is recruited to the cytosolic side of the receptor, and its kinase activity is activated. RIPK1 then interacts with and phosphorylates a related kinase, RIPK3, leading to its activation [59,60,61,62]. RIPK3 then phosphorylates a pseudokinase called MLKL [63], which normally exists as an inactive monomer in the cytosol. Once RIPK3 phosphorylates serine 357 and threonine 358 in human MLKL, or the mouse equivalent serine 345, serine 347, and threonine 349, MLKL forms oligomers and translocates to the plasma membrane, where it disrupts membrane integrity; inflammatory factors including IL-1β and TNF-α are released, resulting in the activation of necrotic cell death [64,65,66,67,68,69,70]. Necroptosis plays a key role in several kinds of disease, including sclerosis, cardiac reperfusion injury, enteritis, organ injury, etc., and the expression levels of RIPK1, RIPK3 and P-MLKL are upregulated in the above diseases [71,72,73,74,75,76,77]. Therefore, this study analyzed the expression of necroptosis factors and inflammatory factors to prove the effect of furosine on the necroptosis pathway and further investigate the toxicity mechanism of furosine in liver tissue. We found that furosine treatment increased the expression levels of RIPK1, RIPK3, P-MLKL, IL-1β, and TNF-α significantly, validating that furosine activated the necroptosis pathway and a downstream inflammatory reaction in the liver. Utilizing siRNA transfection, PLA2-3 was found to regulate the RIPK1/RIPK3/MLKL pathway by affecting the expression of LPC (18:0) in the liver. The combined results confirmed that furosine could activate the *PLA2-3* gene which induced the expression of LPC (18:0), and the latter then led to the activation of downstream necroptosis and inflammation.

In summary, we found that furosine and its metabolic mediator LPC (18:0), regulated by PLA2-3, could lead to liver damage through the activation of cells necroptosis. For the first time, we shed light on furosine toxicity mechanism in the liver in an animal model. Our data might also improve the risk assessment of furosine in foods and provide guidelines to limit furosine levels in food, which is an urgent issue.

## 4. Materials and Methods

### 4.1. Mice Chronic Toxicity Model

ICR mice were purchased from Beijing Vital River Laboratory Animal Technology Co., Ltd. (Beijing, China) with the license number SCXK 2012. Animal experiments were approved by the Ethics Committee of Chinese Academy of Agriculture Sciences (Beijing, china) (Permission number: IAS20181015, 15 October 2018).

Sixty-four ICR mice (20 ± 2 g, 32 male, 32 female) were randomly divided into four groups: control group (without any treatment), 0.1 g/kg furosine group, 0.25 g/kg furosine group, 0.5 g/kg furosine group, with eight male mice and eight female mice in each group. Furosine was purchased from PolyPeptide (Strasbourg, France) at a purity of 95% and was solved in purified water (ddH_2_O). The mice in the treatment groups were gavage-administrated furosine once per day (0.2 mL/mice), in consecutive 42 days. The mice were sacrificed on the 43th day, blood samples were collected from the retro-orbital plexus of mice eyes, and the liver was dissected and frozen in liquid nitrogen for metabonomics analysis and pathological staining.

### 4.2. Histopathologial Test

Liver tissue was isolated and fixed in 4% paraformaldehyde (Solarbio, Beijing, China) for 48 h before the embedding in paraffin and slicing by a slicing machine (Leica, Solms, Germany). The sections were stained with hematoxylin and eosin (HE) for pathological observation and examined under a light microscope (Olympus, Tokyo, Japan). Photographs were taken as indicated (200× magnification).

### 4.3. Biochemical Analysis

Half of the blood was placed into heparin-containing tubes for red blood cells (RBC) separation, and the left was centrifuged to obtain serum for biochemical indicators detection (15 min, 3000 r/min at 4 °C). The protocol of RBC separation was as follows: Saline was added to the blood and, after thoroughly mixing, the samples were centrifuged 2000 r/min centrifugation at 4 °C for 5 min. Then, the sediment was resuspended and washed in Hank’s buffer (without Ca^2+^, Mg^2+^) for three times in the same centrifugation conditions. The resulting sediment was saved as the RBC concentrate and frozen in liquid nitrogen immediately.

The biochemical parameters ALT, AST, ALP, γ-GT, TBil, and LDH were analyzed in mice serum by Elisa kits (Jiancheng, Nanjing, China).

### 4.4. Sample Preparation for Metabonomics Analysis

For sample extraction of mice serum, RBC, and liver tissue, 100 μL serum or RBC or 20 mg tissue was treated with 1.4 mL methanol containing 0.1% formic acid. After ultrasonication for 10 min, the sample solutions were frozen for 1 h at −20 °C for protein precipitation. Then, the samples were centrifuged for 10 min (10,000× *g*, 4 °C), and 1 mL supernatant was transferred into sample vials, which were stored at −20 °C before spectrometry analysis. UPLC–QTOF mass spectrometry analysis and UHPLC–Q-Exactive Orbitrap mass spectrometry analysis were performed. The data were exported into Excel spreadsheets and analyzed by Simca-P for PCA (principle components analysis), PLS-DA (partial least squares discriminant analysis), *t*-test, volcano plot, and VIP plot analysis. The differentially expressed metabolites in control and furosine-treated mice were identified by the following criteria: (1) *p* value < 0.05 (*t* test); (2) VIP value > 1.0.

### 4.5. Quantitative Real-Time PCR (q-PCR) Analysis

We extracted 50–100 ng total RNA from *mouse* liver tissue using the TransZol Up Kit (ET111-01, TransGen Biotech, China). The quantity and concentration of RNA were evaluated in a 1.2% agarose gel and by Nanodrop 2000 (Thermofisher, Waltham, MA, USA). Total RNA was transcribed into cDNA using the High-Capacity cDNA Archive Kit (Applied Biosystems, Foster City, CA, USA) according to the manufacturer’s protocol. Primers used to evaluate the genes phospholipase A2-3 (*PLA2-3*), receptor-interacting proteins-1 (*RIPK-1*), receptor-interacting proteins-3 (*RIPK-3*), mixed lineage kinase domain-like protein (*MLKL*), tumor necrosis factor-α (*TNF-α*), interleukin-1β (*IL-1β*), and *GAPDH* are indicated in Table 1, and *GAPDH* was used as the internal reference to assure equal loadings. Quantitative real-time RT-PCR (qRT-PCR) was performed using 96-well microwell plates in a total volume of 20 μL containing 1 μL template cDNA (10 ng/ μL), 0.5 μL forward primer (10 μM), 0.5 μL reverse primer (10 μM), 10 μL of Universal Master Mix, and 8 μL of RNase-free water (Applied Biosystems, Foster, CA, USA). The qRT-PCR reactions were performed at 94 °C for 30 s, followed by 40 cycles at 94 °C for 5 s and 62 °C for 30 s by using two-step RT-PCR. All qRT-PCR reactions were performed in the ABI 7900 HT system and in triplicate to ensure methodological reproducibility.

### 4.6. Primary Hepatocyte Separation and Culture

ICR mice (18–20 g) were killed and sterilized with 75% ethanol, then the liver tissue was dissected and put in cold PBS buffer. The liver envelope was removed and cut into 1 mm^3^ cubes. The cubes were transferred into a sterile conical flask, trypsin (0.5 mg/L) was added, the flask was incubated under shaking at 37 °C for 15 min, and the supernatant was discarded after 5 min. Collagenase IV (2 mg/L) was added and the samples was incubated under shaking at 37 °C for 25 min, then the digestion was terminated by the addition of DMEM-HG medium containing 10% FBS. After 5 min in static conditions, the supernatant was collected and centrifuged (800 rpm, 5 min). The supernatant was discarded, and the cells were resuspended with DMEM-HG medium. The suspension was filtered (200 mesh), and the filtrate was collected and centrifuged (800 rpm, 5 min). The cells were resuspended in DMEM-HG medium and were centrifuged (800 rpm, 5 min); then the supernatant was discarded. This step repeated three times. The cells were resuspended in DMEM-HG medium containing 10% FBS, 500 U penicillin/streptomycin, and 15 μg/mL of amphotericin B, and the cell concentration was adjusted to 10^5^ cells/mL. The cell suspension was transferred into a sterile flask and cultured in an incubator (37 °C, 5% CO_2_). The medium was changed after 24 h.

### 4.7. Cell Viability Detection

Primary hepatocytes were exposed to furosine at different dosages (0–1000 mg/L) and co-cultured for 48 h, then viability was detected using a CCK-8 kit (Solarbio, Beijing, China), and the proper dosage (associated with about 90% survival rate of primary hepatocytes) of furosine for further experiments was chosen.

### 4.8. Cell siRNA Treatment

On the basis of the primary analysis results, PLA2-3 was chosen as the possible target of furosine, possibly regulating the expression of downstream factors. To further validate the interaction between furosine and these factors, we synthesized PLA2-3 siRNA sequences (Genepharma, Shanghai, China). The cells were seeded into six-well plates for 24 h to obtain 30% confluency. DMEM–DNA–liposome complexes were added to the normal growth medium, and the cells were cultured for 24 h. Furosine treatment started after removing the DMEM–DNA–liposome complex, and the treatment lasted 48 h.

### 4.9. Protein Sample Preparation and Western Blot Detection

Total proteins from liver tissue and primary hepatocytes were extracted using a protein extraction kit (Solarbio, Beijing, China) and measured by a BCA kit (Solarbio, Beijing, China). After heat treatment at 98 °C, the protein samples were loaded onto 12% SDS-polyacrylamide gels and electrophoresis was performed; the proteins were transferred onto nitrocellulose membranes by a Trans-Blot machine (Bio-Rad, Hercules, CA, USA), and the filters were blocked with 2.5% BSA in TBST buffer for 2 h at room temperature (RT). Then, the proteins were probed with primary antibodies for 2.5 h at RT, including anti-β-actin, -PLA2-3, -RIPK-1, -RIPK-3, -MLKL, -P-MLKL (Ser 345), -TNF-α, and -IL-1β antibodies (Abcam, USA). β-actin was used as the internal reference to ensure equal loadings. After washing with PBST buffer (3 × 8 min), the membranes were incubated with secondary antibodies for 1 h at RT and then washed with PBST buffer (4 × 10 min). Finally, the membranes were detected utilizing the ECL reagent and analyzed by Image J software (Rawak Software, Inc. Munich, Germany).

### 4.10. Statistical Analysis

All the data were represented as mean ± SD. Data analysis was performed using GraphPad Prism 6.0 software (GraphPad, San Diego, CA, USA). Statistical analysis was conducted using the Student’s *t*-test and One-Way Analysis of variance (ANOVA); *p* value < 0.05 was considered to indicate a statistically significant difference between the control and treated groups.

## Figures and Tables

**Table 1:** Primer sequence for q-PCR.

Gene Name	Primer Sequences (5′→3′)	
	Forward Primer	Reverse Primer
*PLA2-3*	CCGCAGAGAAGAAGGAGCAGTT	CACGCACAGGAGGCAGAACA
*RIPK-1*	TGAAGCCCACAGCGATTCTT	GCCATCTTTCTCCCCCAAGA
*RIPK-3*	ACATGCATGGTCATGCACACACAT	TTGAGACATCTCTTTTTGGAG
*MLKL*	CCCGAGTTGTTGCAGGAGAT	TCTCCAAGATTCCATCCGCAG
*TNF-α*	GTCCCCAAAGGGATGAGAAGTT	GTTTGCTACGACGTGGGCTACA
*IL-1β*	TGTGAAATGCCACCTTTTGA	GCTCAAAGGTTTGGAAGCAG
*GAPDH*	CAATGAATAGGGCTACAGCA	AGGGAGATGCTCAGTGTTGG

## Supplementary Materials

Supplementary materials can be found at https://www.mdpi.com/1422-0067/20/10/2388/s1.

## Abbreviations

MRP	Maillard reaction product
LPC (18:0)	lysophosphatidylcholine 18:0
PLA2-3	phospholipase A2 gamma
RIPK1	receptor-interacting serine/threonine-protein kinase 1
RIPK3	receptor-interacting serine/threonine-protein kinase 3
MLKL	mixed lineage kinase domain-like protein
