# Supplementary material for: Furosine, a Maillard Reaction Product, Triggers Necroptosis in Hepatocytes by Regulating the RIPK1/RIPK3/MLKL Pathway
Source: Int J Mol Sci. 2019 May 14;20(10):2388. doi: 10.3390/ijms20102388 (PMC6566718; doi:10.3390/ijms20102388)
Supplement: Supplementary file 1 [file ijms-20-02388-s001.zip › ijms-503953-supplementary-final/supplementary figures.pdf]

# Supplementary Figures

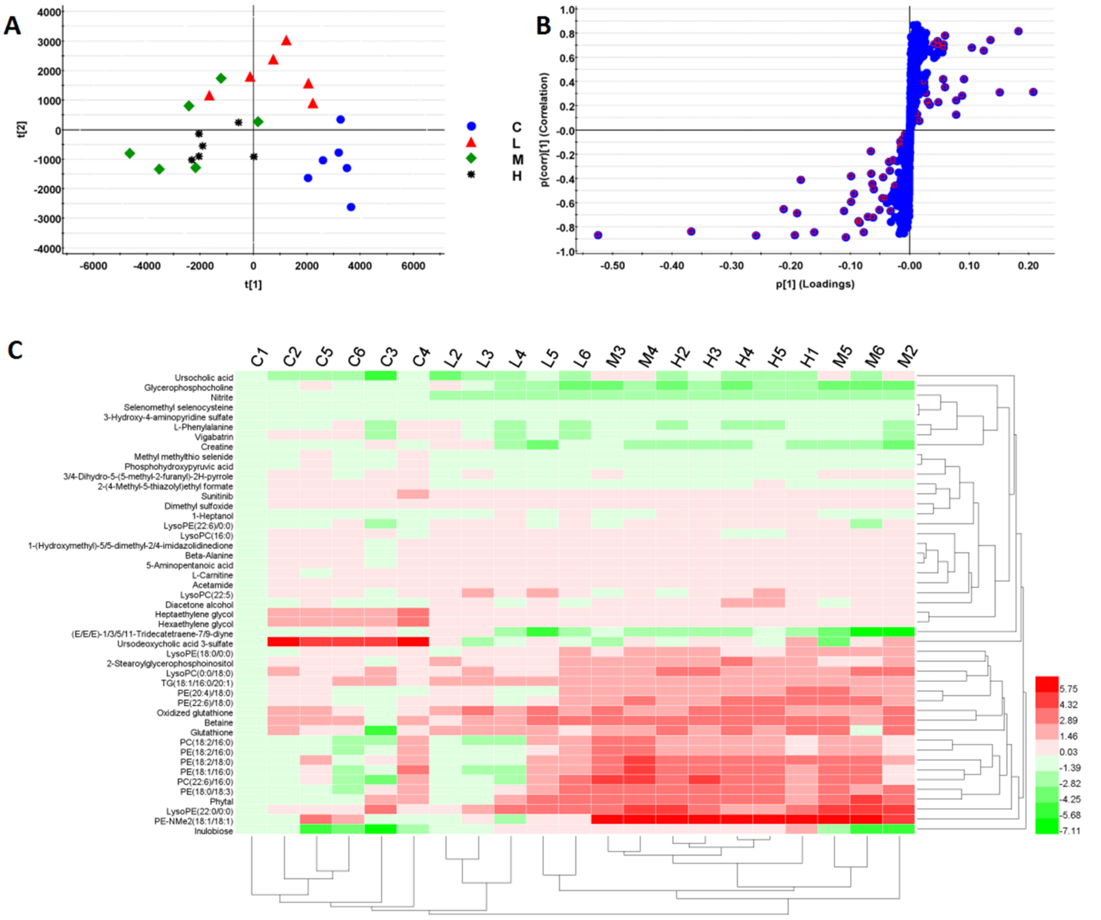


**Figure S1.** OPLS-DA scores plot (**A**), S-plot (**B**) and Heatmap (**C**) of liver tissue metabonomics detection. In **A**, blue dot stands for sample in control group (C), red triangle stands for sample in 0.1 g/kg group (L), green rhombus stands for sample in 0.25 g/kg group (M), black star stands for sample in 0.5 g/kg group (H). In **C**, the red area stands for positive correlation, the green area stands for negative correlation, the darker of the area, the higher of the correlation coefficient.

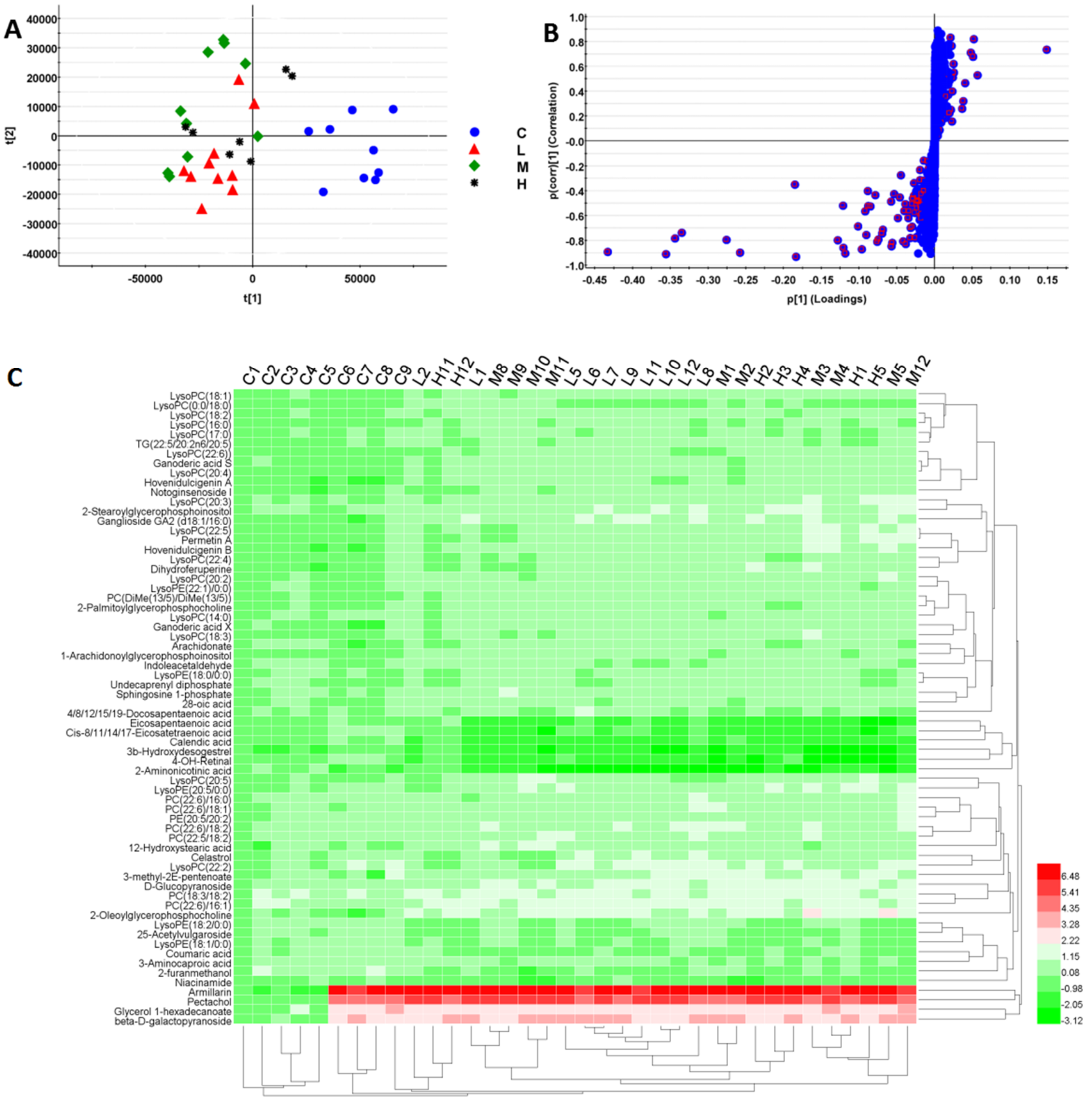


**Figure S2.** OPLS-DA scores plot (**A**), S-plot (**B**) and Heatmap (**C**) of serum metabonomics detection. In A, blue dot stands for sample in control group (C), red triangle stands for sample in 0.1 g/kg group (L), green rhombus stands for sample in 0.25 g/kg group (M), black star stands for sample in 0.5 g/kg group (H). In C, the red area stands for positive correlation, the green area stands for negative correlation, the darker of the area, the higher of the correlation coefficient.

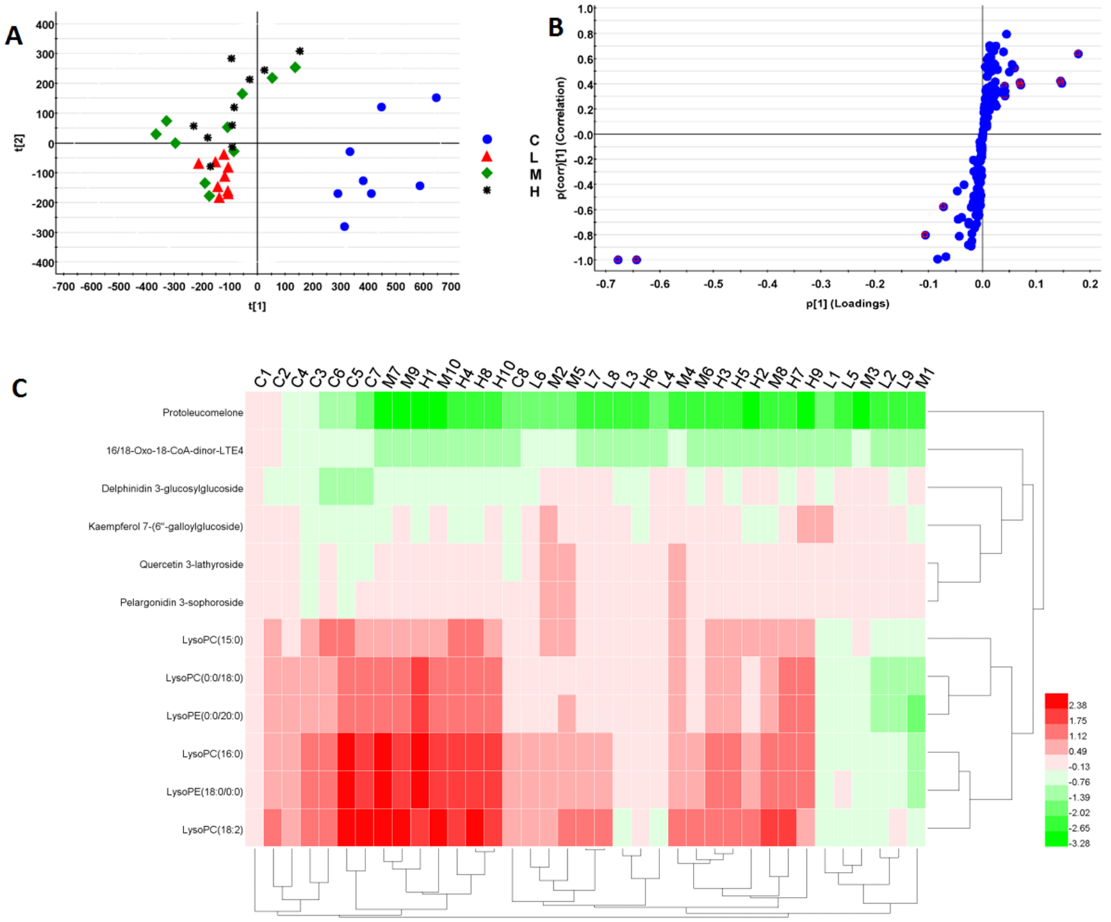


**Figure S3** OPLS-DA scores plot (**A**), S-plot (**B**) and Heatmap (**C**) of RBC metabonomics detection. In **A**, blue dot stands for sample in control group (C), red triangle stands for sample in 0.1 g/kg group (L), green rhombus stands for sample in 0.25 g/kg group (M), black star stands for sample in 0.5 g/kg group (H). In **C**, the red area stands for positive correlation, the green area stands for negative correlation, the darker of the area, the higher of the correlation coefficient.

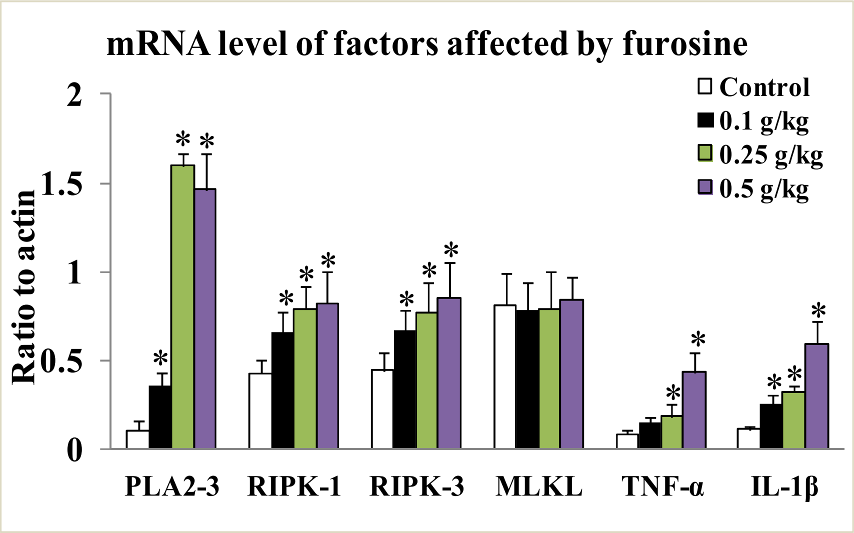


**Figure S4** Expression statistical analysis of *PLA2-3*, *RIPK-1*, *RIPK-3*, *MLKL*, *IL-1β* and *TNF-α* in mRNA level in liver tissue. All the data were represented as mean ± SD, $n = 3$. * Comparing with the control, $p < 0.05$.
